# Supplementary figures and images for: Gene Cascade Finder: A tool for identification of gene cascades and its application in Caenorhabditis elegans
Source: PLoS One. 2019 Sep 10;14(9):e0215187. doi: 10.1371/journal.pone.0215187 (PMC6736238; doi:10.1371/journal.pone.0215187)

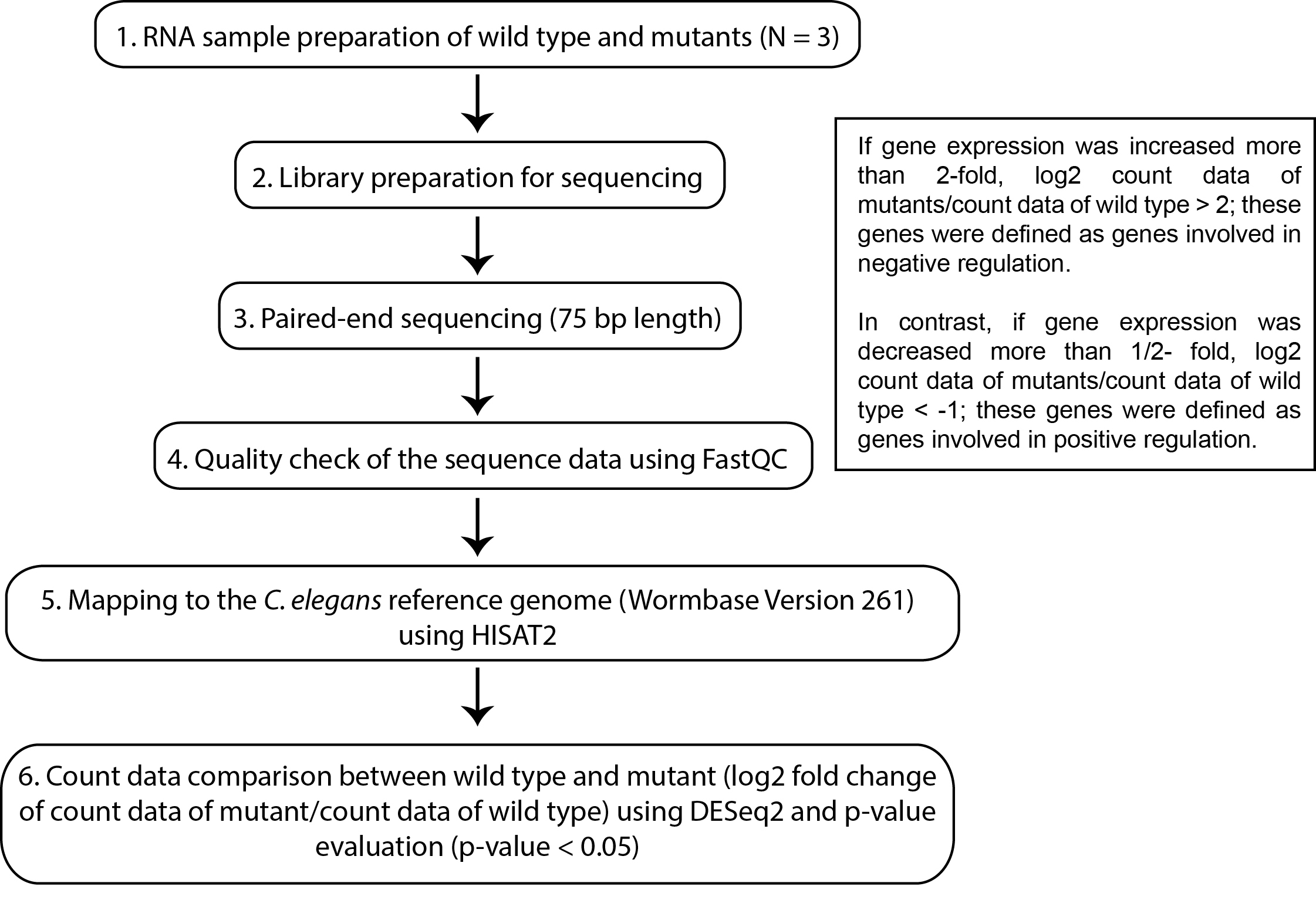

Supplement: S1 Fig — (JPG) [file pone.0215187.s011.jpg]

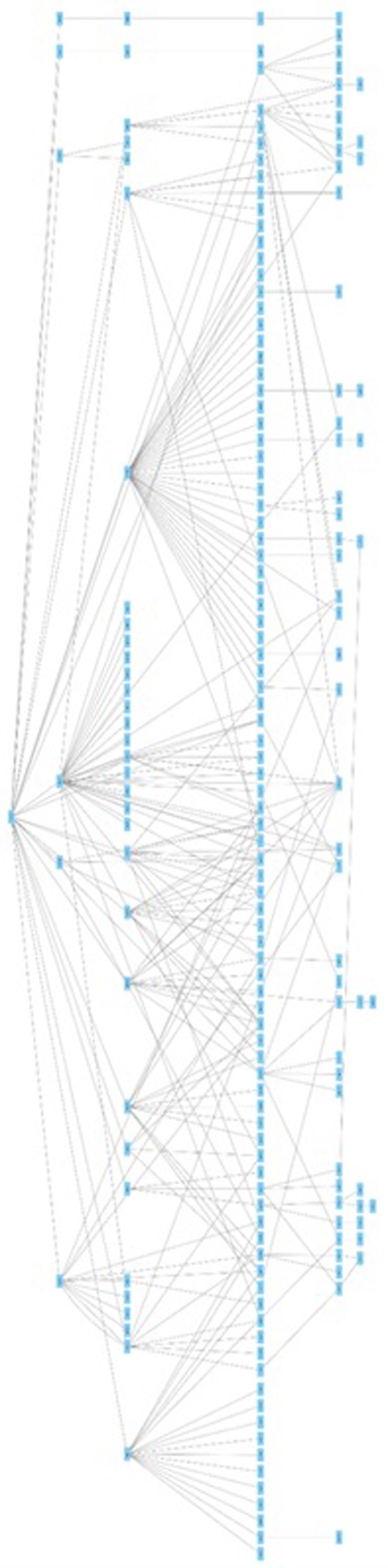

Supplement: S2 Fig — Graphical examples from the GCF software were further processed using Cytoscape, allowing for identification of an output without adding a new input. (JPG) [file pone.0215187.s012.jpg]

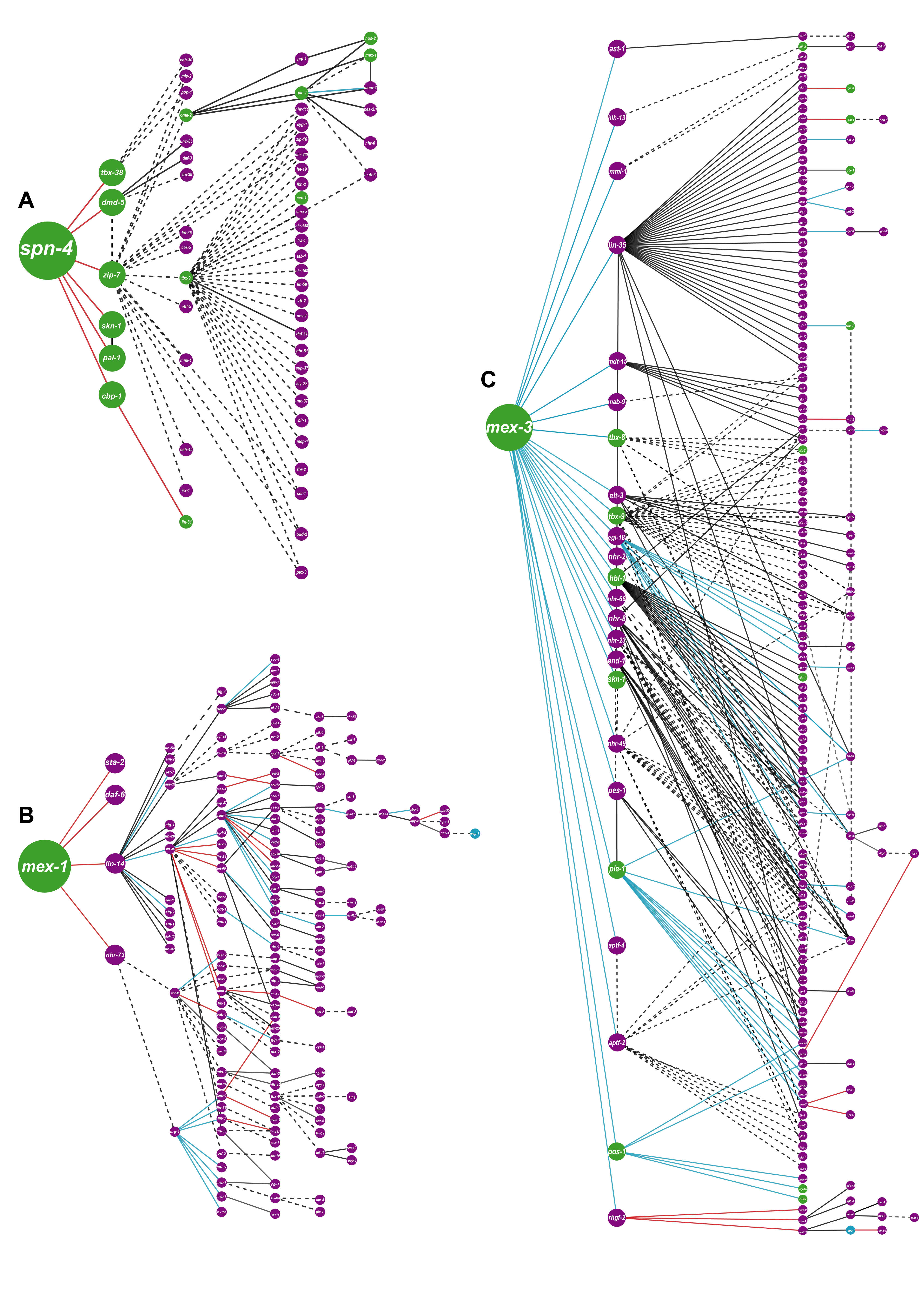

Supplement: S3 Fig — Rendering of each gene cascade was performed using Cytoscape. Nodes indicate each gene in the cascade. Edges indicate the interactions between two genes. Large and intermediate nodes indicate the query genes and the direct target of the query genes, respectively. Other nodes indicate downstream genes. Green nodes indicate genes that are expressed during the early embryonic stage. Purple nodes indicate presumptive early embryonic genes. Red, blue, and black edges indicate positive regulation, negative regulation, and genetic interactions, respectively. Dotted lines indicate protein-protein interactions. (A) spn-4-mediated gene cascade. (B) mex-1-mediated gene cascade. (C) mex-3-mediated gene cascade. (JPG) [file pone.0215187.s013.jpg]
